# Nutritional modulation of oxaliplatin-induced peripheral neuropathy: a serum metabolomics study in colorectal cancer patients

**DOI:** 10.3389/fnut.2026.1781882

**Published:** 2026-03-27

**Authors:** Yun Bian, Haoxing Yuan, Yongjuan Ding, Jianbo Han, Hao Li, Huaneng Xu

**Affiliations:** 1 School of Food Science and Technology, Jiangnan University, Wuxi, China; 2 Department of Pharmacy, Affiliated Hospital of Jiangnan University, Wuxi, China; 3 Department of Neurosurgery, Wujin Hospital Affiliated to Jiangsu University, Changzhou, China

**Keywords:** colorectal cancer, malnutrition, metabolomics, nutritional status, OIPN

## Abstract

The link between nutritional status and chemotherapy toxicity in cancer patients requires further clarification. This study used serum metabolomics to examine how nutritional status affects oxaliplatin-induced peripheral neuropathy (OIPN) in patients with colorectal cancer (CRC). We analyzed samples from 219 CRC patients receiving oxaliplatin-based therapy, grouped by nutritional risk using the Nutritional Risk Screening 2002 (NRS-2002) into two cohorts: malnourished (NRS ≥ 3) and well-nourished (NRS < 3) cohorts. Liquid chromatography-mass spectrometry (LC–MS) and multivariate statistics were used to identify differentially expressed metabolites (DEMs). We found 179 DEMs between OIPN and non-neuropathic controls (CONT), with amino acids and derivatives being the most prevalent. Enrichment analysis pinpointed arginine biosynthesis as a key pathway, exhibiting nutrition-dependent regulation. While L-arginine and ornithine were downregulated and L-glutamine was upregulated in OIPN patients overall, this pattern was reversed in the malnutrition subgroup. Concurrently, arginine pathway enrichment was reduced in malnourished patients. These results indicate that OIPN is associated with significant serum metabolite alterations, primarily affecting amino acid metabolism, which are distinctly modulated by malnutrition. Our findings highlight the role of nutritional status in OIPN occurrence and may provide a basis for future research into targeted nutritional support to alleviate this neurotoxicity in CRC patients, although confirmatory studies are needed.

## 1 Introduction

The nutritional status of cancer patients plays a pivotal role in determining their quality of life and therapeutic outcomes. Suboptimal nutritional status can exert detrimental effects throughout the entire treatment continuum, manifesting as impaired immune defenses, heightened susceptibility to infections, and compromised treatment tolerance. Nutritional deterioration may occur at any stage during cancer diagnosis and treatment, with the peri-chemotherapy phase presenting particular vulnerability. Emerging evidence indicates that malnutrition during this critical period demonstrates significant associations with disease progression, adverse chemotherapy responses, and diminished prognostic outcomes.

Colorectal cancer (CRC) is a malignant tumor affecting the digestive tract, as reported in the 2018 China Cancer Statistics Report (1). The incidence and mortality of CRC in China ranked third and fifth, respectively, among all malignant tumors. Oxaliplatin is a third-generation platinum-based antineoplastic drug (2, 3) that serves as a first-line treatment for CRC and other digestive system tumors. One of the primary side effects associated with oxaliplatin is oxaliplatin-induced peripheral neurotoxicity (OIPN). OIPN is characterized by symptoms such as sensitivity to cold, discomfort in the throat, difficulty swallowing cold liquids, and muscle cramps (4, 5). These side effects can negatively impact the efficacy of chemotherapy and the overall quality of life for patients (6). At present, there are no guidelines, either domestically or internationally, recommending drugs that can effectively prevent and treat peripheral neurotoxicity. The “Prevention and Management of Chemotherapy-Induced Peripheral Neuropathy in Survivors of Adult Cancers: ASCO, 2020 Guideline Update” comprehensively evaluates the quality of evidence and the strength of recommendations for some clinical preventive measures and preventive drugs. It also summarizes areas where these recommendations do not apply (7). The guidelines do not provide effective solutions while standardizing clinical prevention and treatment measures and drugs and have limited clinical effects.

Inadequate nutritional status adversely impacts the entire cancer treatment course, leading to weakened immune defenses, increased infection risk, and reduced treatment tolerance (8–10). Despite their clinical significance, critical gaps remain in understanding how nutrition influences oxaliplatin-induced peripheral neuropathy (OIPN). This study uses longitudinal metabolomic profiling of serial serum specimens from CRC patients receiving oxaliplatin-based regimens to elucidate nutrition–metabolism–neurotoxicity interactions. Through integrated analysis of pharmacometabolic pathways and nutritional biomarkers, we aim to (1) systematically investigate the interplay between dynamic nutritional status and OIPN pathogenesis, (2) identify nutritionally driven metabolic determinants that contribute to interindividual variability in neurotoxic manifestations, and (3) establish nutrition-focused predictive models for assessing the risk of OIPN. The mechanistic insights into nutrient-sensitive metabolic reprogramming could guide the creation of precision nutritional intervention protocols tailored for malnourished cancer populations, ultimately striving to develop prevention strategies that are informed by these mechanisms to reduce the risk of neurological complications induced by chemotherapy.

## 2 Materials and methods

### 2.1 Patient recruitment

A total of 219 patients with colorectal cancer admitted to the Affiliated Hospital of Jiangnan University in Wuxi from July 2022 to May 2023 were selected. Of these, 65.75% (144 cases) developed OIPN and were classified as the OIPN group, while 34.25% (75 cases) did not develop OIPN and were classified as the non-OIPN (CONT) group. This study was conducted in accordance with the guiding principles outlined in the Declaration of Helsinki. All procedures involving human subjects/patients were approved by the Medical Ethics Committee of the Affiliated Hospital of Jiangnan University (ethics approval number: LS2022080). The treatment regimen FOLFOX, which inlcudes oxaliplatin, calcium folinate, and 5-fluorouracil, was administered, and the occurrence of OIPN after chemotherapy was observed. In this trial, patients who had undergone curative-intent colorectal cancer resection were scheduled to receive 12 cycles of FOLFOX, which included 135 mg/m^2^ oxaliplatin every 2 weeks.

### 2.2 Clinical data collection

Demographic and clinical characteristics of all participants were recorded, including age, sex, height, and weight. Body mass index (BMI) was calculated as weight in kilograms divided by the square of height in meters (kg/m^2^). Nutritional risk was assessed using the Nutritional Risk Screening 2002 (NRS-2002) tool. Patients with an NRS-2002 score ≥ 3 were classified as being at nutritional risk (malnourished), while those with a score < 3 were considered well-nourished.

OIPN was evaluated by trained clinicians after each chemotherapy cycle. Neurological assessment included routine physical examination and grading according to the following criteria. Patients were asked to independently complete the patient neurotoxicity questionnaire to assess OIPN according to Common Terminology Criteria for Adverse Events (CTCAEs) version 6.0, with some adjustments for clinical need.

Grade I: Loss of deep tendon reflexes or paresthesia, with no impact on physical function; symptoms are either absent or detectable only upon examination.

Grade II: Needling sensation or other paresthesia causing mild symptoms that affect physical function but do not interfere with daily activities.

Grade III: Abnormal sensory changes that mildly affect daily life, requiring assistive devices such as walking sticks or wheelchairs.

Grade IV: Disability that is life-threatening.

When calculating the overall occurrence rates of CTCAEs, we used the highest grade of OIPN observed during any of the chemotherapy cycles for each patient.

The severity grade and frequency of neuropathic symptoms (e.g., duration and daily attacks) were recorded.

### 2.3 Blood sample collection and processing

A 2 mL of sample of fasting venous blood was collected prior to the first cycle of chemotherapy (baseline). An aliquot of the blood sample was immediately analyzed using an automated hematology analyzer to determine serum albumin levels, peripheral blood lymphocyte count, and hemoglobin concentration. Height and weight were recorded, and body mass index (BMI) was calculated. The remaining blood sample was centrifuged at 4 °C to separate the serum, which was then aliquoted and stored at −80 °C until subsequent metabolomic analysis, which was performed by Panomix Biomedical Tech Co., Ltd. (Suzhou, China).

### 2.4 Serum metabolomic data preprocessing and metabolite identification

The raw data were first converted to mz XML format by MS Convert iProteo Wizard software package (v3.0.8789) (11) and processed using R XCMS (v3.12.0) for feature detection (12), retention time correction, and alignment. Key parameter settings were set as follows: ppm = 15, peak width = c (5,30), mzdiff = 0.01, method = cent Wave. The batch effect was then eliminated by correcting the data based on QC samples. Metabolites with RSD > 30% in QC samples were filtered and then used for subsequent data analysis. The metabolites were identified by accurate mass and MS/MS data, which were matched with HMDB^1^ (13), massbank^2^ (14), KEGG^3^ (15), LipidMaps^4^ (16), mzcloud^5^ (17), and the metabolite database built by Panomix Biomedical Tech Co., Ltd. (Suzhou, China). The molecular weight of metabolites was determined according to the m/z (mass-to-charge ratio) of parent ions in MS data. The molecular formula was predicted by ppm (parts per million) and adduct ion, and then matched with the database to realize MS identification of metabolites. At the same time, the MS/MS data from the quantitative table of MS/MS data were matched with the fragment ions and other information of each metabolite in the database, to realize the MS/MS identification of metabolites.

### 2.5 Data analysis

Two different multivariate statistical analysis models, unsupervised and supervised, were applied to discriminate the groups (PCA; PLS-DA; OPLS-DA) by the R ropls (v1.22.0) package. The statistical significance of the *p*-value was obtained by a statistical test between groups. Finally, combined with *p*-value, VIP (OPLS-DA variable projection importance), and FC (multiple of difference between groups) screened biomarker metabolites. By default, when the *p*-value < 0.05 and the VIP value >1, we consider the metabolite to have significant differential expression.

### 2.6 Pathway analysis

Differential metabolites were subjected to pathway analysis by Metabolomics Analyst, which combines results from powerful pathway enrichment analysis with the pathway topology analysis. The identified metabolites in metabolomics were then mapped to KEGG pathways for biological interpretation of higher-level systemic functions. The metabolites and their corresponding pathways were visualized using the KEGG Mapper tool.

### 2.7 Statistical analysis

R software and related packages (ROPLS, Metabolomics Analyst), SPSS (version 26.0), and Graph Pad Prism (version 9.2) were used to analyze the data and plot. The measurement data in line with normal distribution were expressed by (*χ* ± s); the comparison between groups was expressed by two independent samples T-test; the enumeration data were expressed by [*n* (%)]; the difference between groups was expressed by chi-square test; the difference was statistically significant when the *p*-value of < 0.05.

## 3 Results

### 3.1 Nutritional modulation of OIPN occurrence

A total of 219 patients with colorectal cancer were enrolled, including 149 males and 70 females, with an age range of 31 to 77 years (mean ± SD: 62.27 ± 9.30 years). Tumor metastasis was present in 126 patients in the OIPN group and 66 patients in the CONT group. No significant differences were found between the two groups regarding gender, age, tumor metastasis, or BMI. However, there was a significant difference in the incidence of complications between the two groups (*p* < 0.05) (Table 1). After oxaliplatin chemotherapy, 100 patients (69%) had OIPN lasting for 0–10 min (Table 2), 112 patients (78%) had daily attacks (Table 3), and most patients (59%) had severity grade II (Table 4). The incidence of NRS-2002 ≥ 3 in the OIPN group (90.97%) was significantly higher than that in the CONT group (77.33%), with a statistically significant difference between the groups (*p* = 0.005). Abnormal (low) albumin levels in the OIPN group were 36.25 ± 2.66 g/L, showing a statistically significant intergroup difference (*p* = 0.000). Similarly, abnormal (low) hemoglobin levels in the OIPN group were 105.14 ± 12.27 g/L, with a significant difference between groups (*p* = 0.022) (Table 5).

**Table 1:** General data on patients with colorectal cancer.

Project	OIPN (*n* = 144)	CONT (*n* = 75)	*T/χ^2^* value	*p-*value
Sex [case (%)]			0.778	0.768
Male	97 (67.36)	52 (69.33)		
Female	47 (32.64)	23 (30.67)		
Age ( $\bar{\chi }\pm s$ , years)			2.086	0.092
<50	45.50 ± 6.67	44.10 ± 3.90		
[50,70)	60.97 ± 5.78	61.22 ± 5.25		
≥70	72.60 ± 2.43	73.07 ± 2.30		
BMI ( $\bar{\chi }\pm s$ , kg/m^2^)			−0.549	0.711
BMI<18.5	17.56 ± 0.81	16.79 ± 0.76		
18.5 ≤ BMI ≤ 24	21.19 ± 1.47	21.49 ± 1.62		
BMI>24	25.47 ± 1.19	25.88 ± 2.09		

**Table 2:** Duration time of OIPN.

Duration	Cases (%)
0–10 min	100 (69)
10–30 min	17 (12)
31–60 min	6 (4)
1–2 h	4 (3)
>2 h	7 (5)
≥ Half a day	10 (7)

**Table 3:** Frequency of OIPN.

Frequency	Cases (%)
Once	29 (20)
Every day	112 (78)
The whole chemotherapy cycle	3 (2)

**Table 4:** OIPN severity classification.

Classification of peripheral neurotoxicity	Cases (%)
Grade I	28 (19)
Grade II	85 (59)
Grade III	30 (21)
Grade IV	1 (1)

**Table 5:** Comparison of nutritional status in patients with colorectal cancer.

Project	OIPN (*n* = 144)	CONT (*n* = 75)	*T/χ^2^* value	*p-*value
NRS-2002 [Example (%)]			0.187	*0.005
≥3	131 (90.97)	58 (77.33)		
<3	13 (9.03)	17 (22.67)		
Albumin ( $\bar{\chi }\pm s$ , G/L)			−8.994	***0.000
Abnormal (low)	36.25 ± 2.66	35.22 ± 3.93		
Normal	42.19 ± 1.70	41.90 ± 1.26		
Peripheral blood lymphocyte count ( $\bar{\chi }\pm s$ × 10 ^ 9/L)			−2.141	0.094
Abnormal	0.90 ± 0.19	0.89 ± 0.07		
Normal	1.63 ± 0.45	1.59 ± 0.37		
Hemoglobin ( $\bar{\chi }\pm s$ , G/L)			−2.763	*0.022
Abnormal (low)	105.14 ± 12.27	103.32 ± 17.19		
Normal	134.23 ± 8.87	133.83 ± 10.67		

*p < 0.05 and ***p < 0.001.

### 3.2 Metabolite profiling and classification

**Figure 1:**
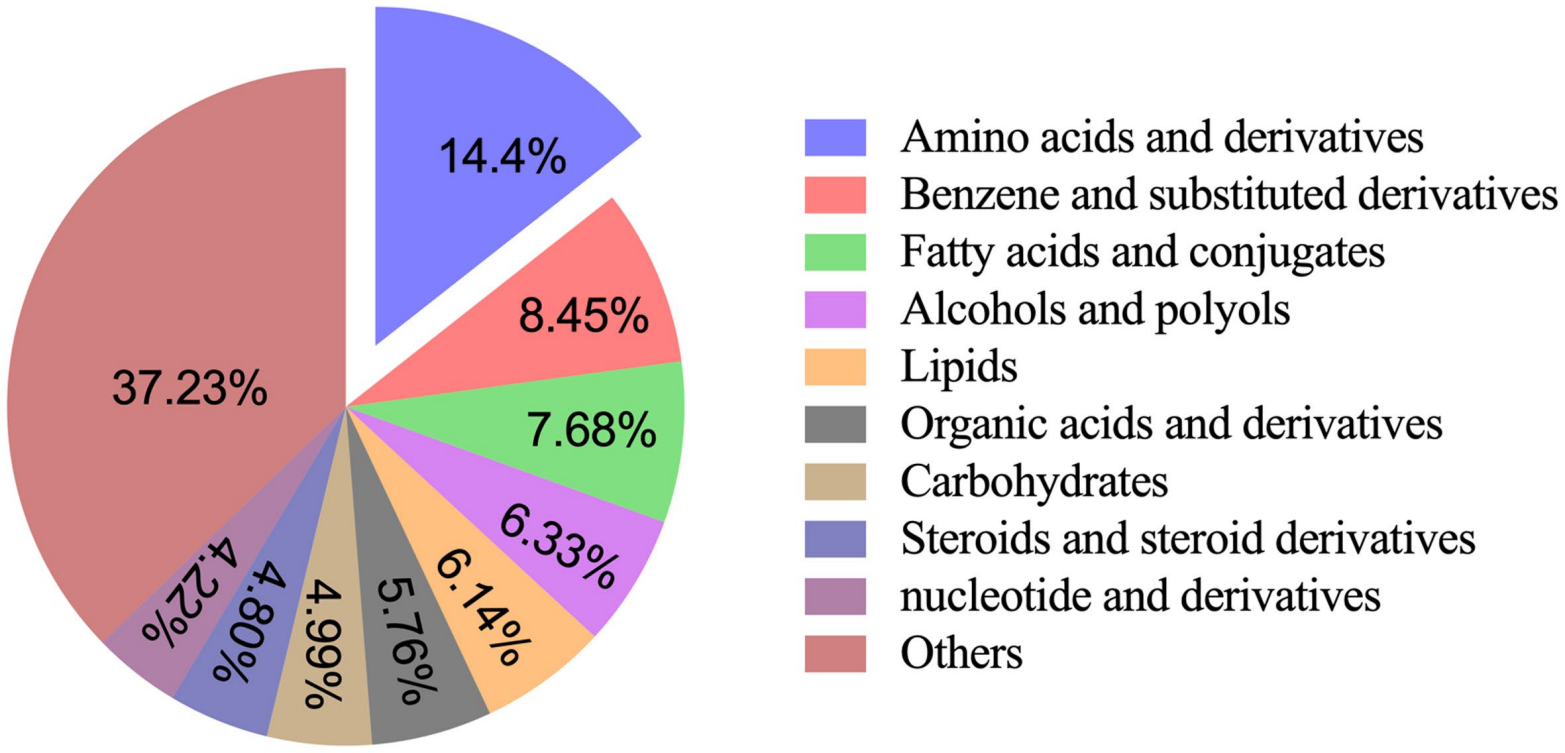
Metabolite classification proportion (%).

Serum samples from colorectal cancer patients were subjected to metabolomic analysis. Based on nutritional risk screening (NRS-2002), participants were categorized into three cohorts: the overall cohort (*n* = 219, comprising 144 OIPN and 75 CONT patients), the malnutrition group (NRS-2002 ≥ 3, *n* = 189, including 131 OIPN and 58 CONT), and the well-nourished group (NRS-2002 < 3, *n* = 30, including 13 OIPN and 17 CONT). Figure 1 presents representative total ion chromatograms for OIPN versus CONT samples in both positive and negative ion modes. After functional analysis of these metabolites and exclusion of ion peaks that did not meet qualitative thresholds, a total of 521 metabolites were reliably identified. Of these, 315 and 206 were detected in the positive- and negative-ion modes, respectively. These metabolites were classified into 22 categories. The five most abundant categories were: amino acids and derivatives (75, 14.40%), benzene and substituted derivatives (44, 8.45%), fatty acids and conjugates (40, 7.68%), alcohols and polyols (33, 6.33%), and lipids (32, 6.14%).

### 3.3 Multivariate statistical analysis of metabolites

Principal component analysis (PCA) was performed on the metabolite profiles from the overall sample set (OIPN vs. CONT), nutritional subgroups (well-nourished and malnourished), and quality control (QC) samples. The first two principal components (PC1 and PC2) accounted for 47.4 and 37.8% of the total variance in positive and negative ion modes, respectively. PCA showed clear separation between OIPN and CONT groups along PC1, with OIPN patients exhibiting greater metabolomic variation. Distinct clustering was also observed between the groups in different nutritional states.

**Figure 2:**
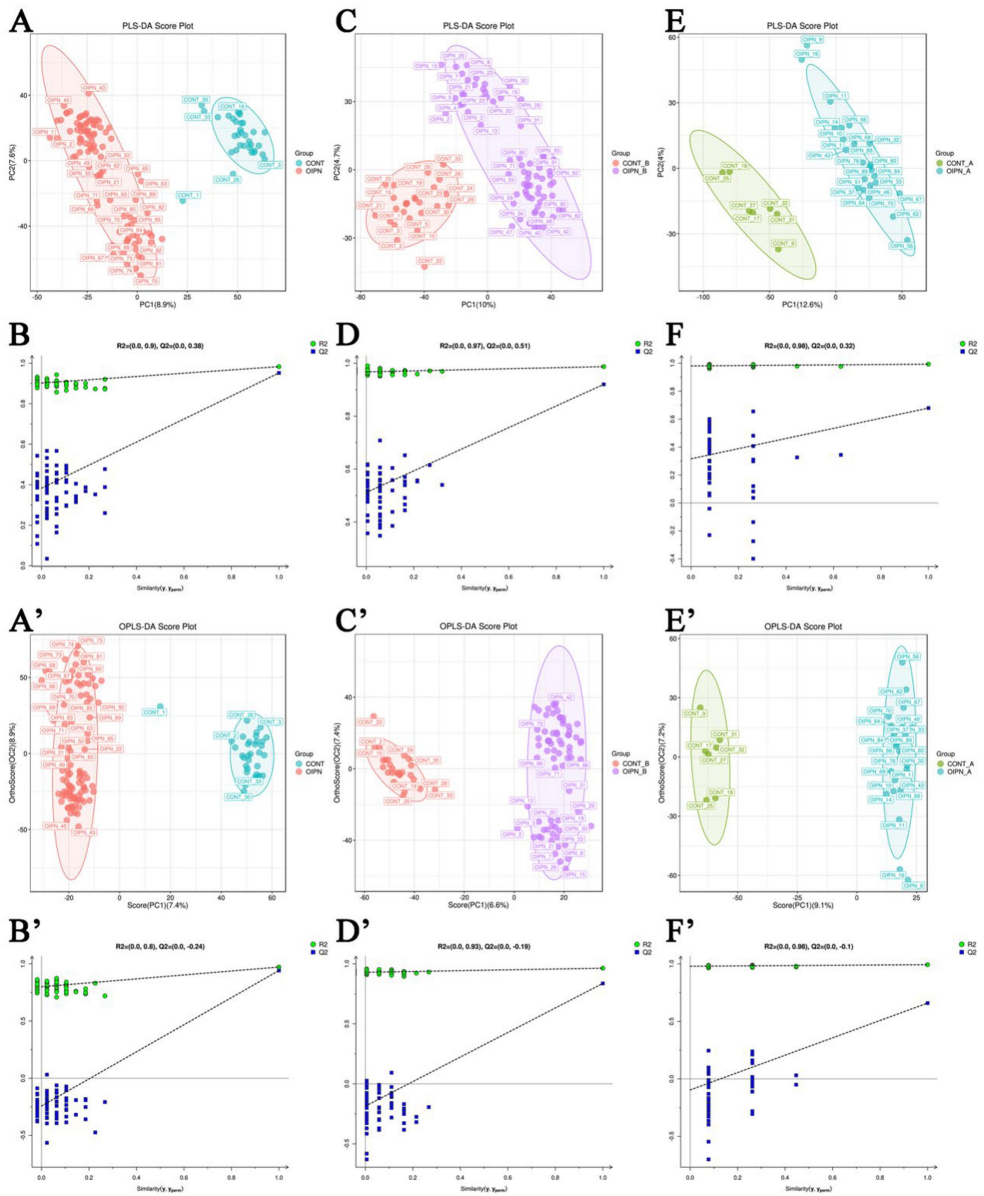
**(A,B)** Permutation sequencing results of PLS-DA mode in positive ion (+) mode in the OIPN vs. CONT and QC for the whole group. **(C,D)** PLS-DA results in the malnutrition group. **(E,F)** PLS-DA results in the well-nourished group; **(A′,B′)** OPLS-DA results in the whole group in positive (+) ion mode. **(C′,D′)** OPLS-DA results in the malnutrition group. **(E′,F′)** OPLS-DA results in the well-nourished group. Correction for multiple testing was applied when identifying differentially expressed metabolites.

To further assess data reliability, partial least squares-discriminant analysis (PLS-DA) was applied. Permutation tests confirmed the absence of overfitting, supporting the validity and reproducibility of the model. Hierarchical clustering analysis likewise demonstrated clear separation between OIPN and CONT metabolites across all nutritional conditions (Figures 2A–F).

Compared with PLS-DA, orthogonal PLS-DA (OPLS-DA) reduces model complexity while preserving predictive performance and enhancing interpretability. OPLS-DA score plots revealed well-separated clusters between OIPN and CONT groups in the overall cohort as well as in each nutritional subgroup, with no overlap (Figures 2Aʹ–Fʹ), indicating distinct metabolite profiles between OIPN and CONT patients irrespective of nutritional status.

### 3.4 Differentially expressed metabolite identification and analysis

**Figure 3:**
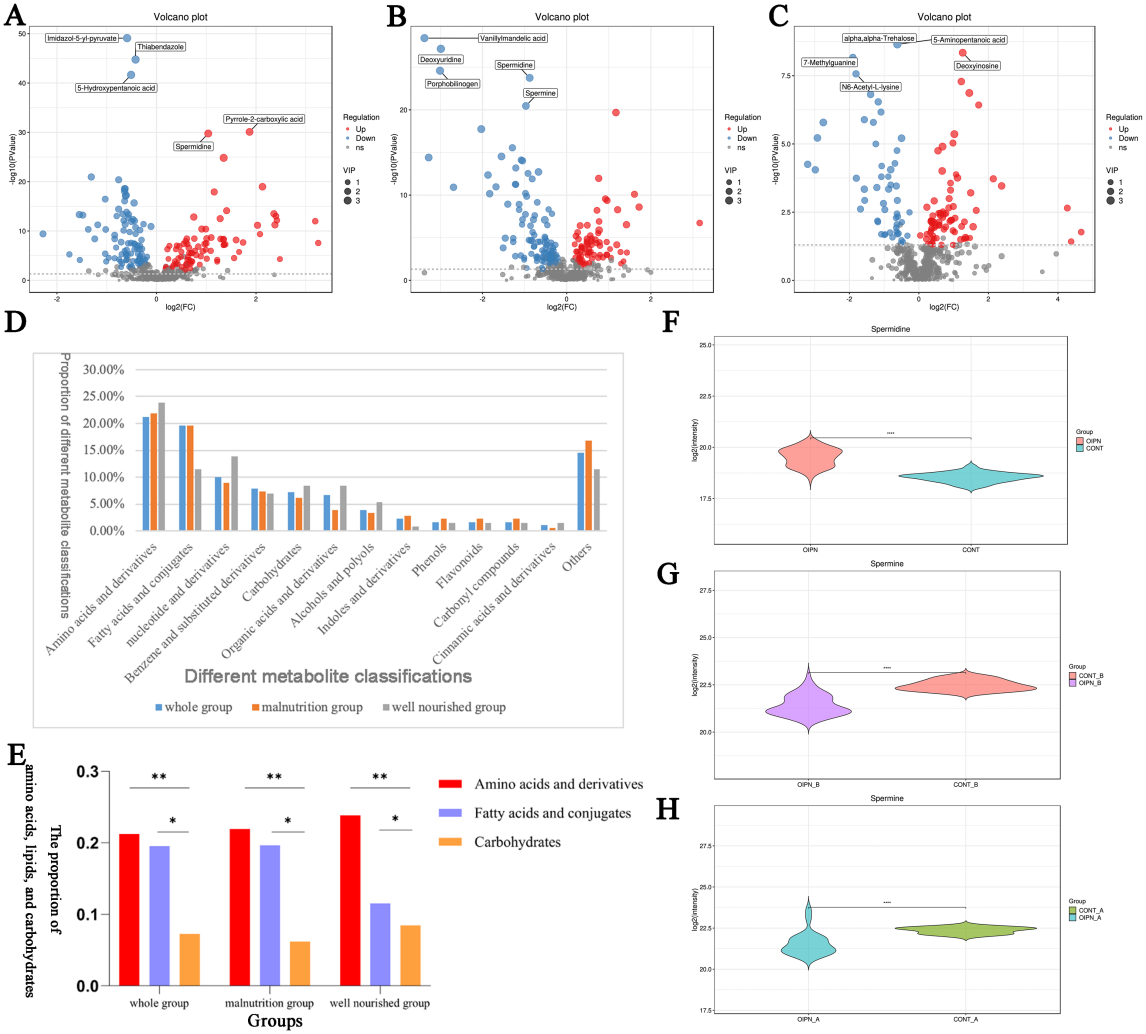
**(A–C)** Distribution and change trend of differential metabolites of OIPN vs. CONT in the whole group **(A)**, malnutrition group **(B)**, and well-nourished group **(C)**. **(D)** Statistics of 13 DEMs of OIPN vs. CONT for different comparisons in the whole group, malnutrition group, and well-nourished group. **(E)** Statistics of amino acids, lipids, and carbohydrates from OIPN vs. CONT in different comparisons in the whole group, malnutrition group, and well-nourished group. **(F–H)** Significant differences in the quantitation of spermidine in the whole group **(F)**, malnutrition group **(G)**, and well-nourished group **(H)**. *p < 0.05, ** *p* < 0.01, and *** *p* < 0.001, *t*-test.

Differentially expressed metabolites (DEMs) were identified between OIPN and CONT groups using established criteria (VIP > 1, *p* ≤ 0.05; two-sample t-test), revealing distinct metabolic profiles in the overall cohort. A total of 179 DEMs were detected, including 82 up-regulated and 97 downregulated metabolites (Figure 3A). Following stratification by nutritional status, 178 DEMs were identified in the malnutrition group (75 up-regulated, 103 downregulated; Figure 3B) and 130 DEMs in the well-nourished group (77 up-regulated, 53 downregulated; Figure 3C). These DEMs spanned diverse chemical classes—such as alcohols and polyols, amines, amino acids and derivatives, benzene and substituted derivatives, carbohydrates, esters, flavonoids, and organic acids and derivatives—with amino acids and their derivatives being the most abundant, followed by fatty acids and conjugates, benzene and substituted derivatives, carbohydrates, organic acids and derivatives, and nucleotides and derivatives (Figure 3D).

Among the differential metabolites identified, the proportions and comparative analysis of three major nutritional categories—amino acids, lipids, and carbohydrates—are summarized in Figure 3E. Differential amino acid metabolites accounted for the highest proportion across all groups. Beyond their fundamental role in protein synthesis, amino acids perform vital physiological functions, and their altered abundance was statistically significant relative to lipids and carbohydrates.

Further analysis confirmed that amino acids and their derivatives constituted the largest category of differentially expressed metabolites. Their significant involvement in specific metabolic pathways highlights that these amino acids serve not only as fundamental building blocks for proteins but also as key precursors to a wide range of biologically and physiologically important molecules (18). Among the markedly altered amino acids and derivatives, spermidine was prominently upregulated in the overall cohort. Quantitative analysis revealed highly significant differences in spermidine levels between OIPN and CONT groups across all nutritional strata: in the whole cohort (Figure 3F, *p* < 0.001), the malnutrition group (Figure 3G, *p* < 0.001), and the well-nourished group (Figure 3H, *p* < 0.001). This consistent upregulation, observed in the context of an enriched arginine metabolic pathway, positions spermidine as a potentially important metabolic node in the development of neuropathy.

### 3.5 Functional enrichment analysis of differentially expressed metabolites

**Figure 4:**
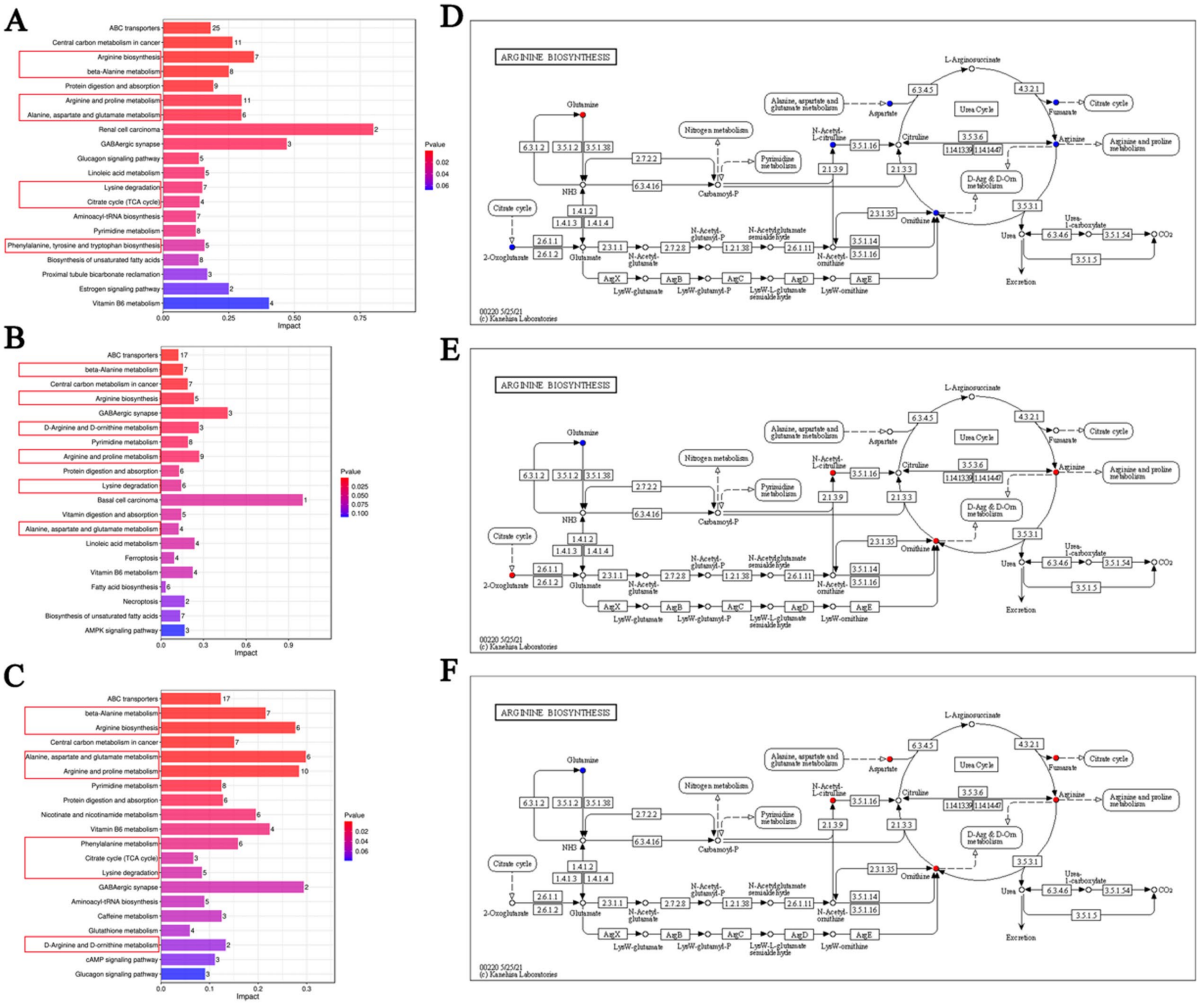
**(A–C)** Histogram of influence factors in OIPN vs. CONT samples on the metabolic pathway in the whole group **(A)**, malnutrition group **(B)**, and well-nourished group **(C)**, respectively. **(D–F)** Analysis of the arginine metabolic pathway in the whole group **(D)**, malnutrition group **(E)**, and well-nourished group **(F)**, respectively.

Functional enrichment analysis using the Kyoto Encyclopedia of Genes and Genomes (KEGG) revealed the top 20 metabolic pathways across the overall cohort and within different nutritional states. Amino acid-related pathways were the most frequently represented among these top pathways, appearing in seven pathways for OIPN vs. CONT in the whole group (Figure 4A), six in the malnutrition group (Figure 4B), and eight in the well-nourished group (Figure 4C). This was followed by organic system metabolic pathways involving the nervous, digestive, intestinal, and endocrine systems, which accounted for five pathways in the whole group and three each in the malnutrition and well-nourished groups. Additionally, membrane transport and conduction pathways were also prominent, comprising one pathway in the whole group and two each in the two nutritional subgroups (Figures 4A–C).

Analysis of shared metabolic pathways across the categorized samples revealed that amino acid metabolism was the most consistently represented. Given that amino acid metabolism is sensitive to physiological stress, infection, and nutritional changes, these pathways likely reflect the patient’s nutritional status and appear to play a central role in the pathogenesis of OIPN across different nutritional states.

Notably, the “Arginine biosynthesis” pathway (hsa00220) was repeatedly identified as a key pathway. Its enrichment is consistent with the upregulation of spermidine in the overall OIPN group, as arginine serves as a substrate for spermidine synthesis via the ornithine cycle. Furthermore, this pathway was significantly less enriched in malnourished patients than in well-nourished individuals, suggesting that its activity is modulated by nutritional status.

Arginine is a functionally versatile amino acid, serving as a precursor for nitric oxide, phosphocreatine, and polyamines, and is involved in gene regulation. Its dysregulated metabolism is increasingly recognized as a hallmark of cancer metabolism and is closely linked to colorectal cancer progression (19). Studies indicate that modulation of arginine biosynthesis can suppress tumor proliferation and metastasis. Notably, research reveals that in the context of aging and tissue injury, such as chemotherapy-induced intestinal damage, arginine metabolism can be adaptively redirected towards polyamine synthesis to support epithelial regeneration and alleviate proteostasis stress (20). This metabolic plasticity suggests that targeted nutritional strategies, including arginine or polyamine supplementation, could be used to improve tissue repair and mitigate treatment-related side effects in aged individuals. Clinically, arginine supplementation has been shown to improve nutritional status, reduce complications, and enhance immunity in cancer patients (20), highlighting its potential therapeutic value when applied within a carefully balanced framework that considers both its regenerative benefits and oncological risks.

In this study, the arginine biosynthesis pathway contained seven differential metabolites. Comparison with enrichment analysis results showed one upregulated metabolite (L-glutamine) and six down-regulated metabolites: fumaric acid, L-aspartic acid, L-arginine, ornithine, oxoglutaric acid, and N-*α*-acetyl citrulline (Figures 4D–F). This pattern further underscores the close interaction between arginine metabolism, host nutrition, and OIPN development.

Based on these results, it can be hypothesized that the arginine biosynthesis pathway may be related to oxaliplatin metabolism and side effects in colorectal cancer patients. This pathway could be a key factor for further exploration of OIPN occurrence. Simultaneously, investigating the internal correlation of OIPN from the perspective of different patient nutritional states may provide a basis for precise nutritional support, especially for malnourished cancer patients, aiming to prevent and treat chemotherapy-induced side effects.

## 4 Discussion

Understanding the metabolic links between nutritional status and oxaliplatin-induced peripheral neuropathy (OIPN) in colorectal cancer patients presents a key challenge for improving its prevention and treatment. In this study, we performed serum metabolomic profiling of patients receiving oxaliplatin, stratified by nutritional status into malnourished and well-nourished groups. Our goal was to characterize OIPN-associated metabolic alterations and to examine how nutritional state modulates these changes. We observed significant differential accumulation of metabolites between OIPN and CONT groups, with the most pronounced downregulation occurring in malnourished OIPN patients, underscoring the clear influence of malnutrition on OIPN development.

Notably, among the differentially expressed metabolites (DEMs), amino acids—followed by lipids and carbohydrates—were the most prominently altered. For example, L-proline was downregulated in OIPN patients overall. Given that proline and its derivatives exhibit anti-inflammatory and antioxidant activities (21–23), its reduction may be mechanistically relevant to OIPN pathogenesis.

Equally important, spermidine was up-regulated in the overall OIPN cohort, a change linked to the enriched arginine metabolic pathway. This suggests OIPN may activate arginine metabolism to promote spermidine synthesis. Spermidine, a neuroprotective polyamine, enhances autophagy, reduces inflammation, and supports mitochondrial function, highlighting its potential role in mitigating neurological injury (24, 25). In contrast, malnourished patients exhibited inhibition of the arginine biosynthesis pathway, accompanied by a less pronounced upregulation of spermidine relative to the other two groups and the absence of reversal in the directional changes in arginine and ornithine. This metabolic pattern suggests that malnutrition may compromise the amino acid pool, restrict substrate conversion, and impair the host’s capacity to counteract OIPN, potentially exacerbating its neurotoxic effects. The mechanism of OIPN is not yet clear. Especially, the link between nutritional status and OIPN in cancer patients has just begun to attract people’s attention. This study found that OIPN is associated with significant serum metabolite alterations, primarily in arginine/spermidine metabolism. These findings suggest potential associations warranting further investigation and may provide preliminary insights into possible targets for future nutritional interventions. However, these observations are exploratory and hypothesis-generating, and causal relationships cannot be inferred from the current data. Spermidine, a key polyamine, improves protein production efficiency by modifying the eIF5A protein through hypusination. This modified eIF5A helps resolve stalling during the translation of polyproline sequences and enhances the final step of protein synthesis (26). Additionally, polyamines such as spermidine play vital roles in cell growth, energy production in mitochondria, and immune system functions (27). The recent study (28) also revealed that polyamines such as spermidine could sustain epithelial regeneration in aging intestines by modulating protein homeostasis.

Several limitations of this study should be acknowledged. This study is a real-world investigation, with one of its primary aims being to reveal the impact of pre-chemotherapy nutritional status on the occurrence of OIPN in colorectal cancer patients. Although it demonstrates that baseline nutritional status may serve as a risk factor for OIPN during chemotherapy, the mechanisms of action of nutritional factors on OIPN require further investigation, and our findings represent an exploratory step toward understanding potential metabolic pathways. Due to the substantial imbalance in sample sizes between nutritional subgroups (131 malnourished OIPN patients versus only 13 well-nourished OIPN patients), we were unable to perform direct metabolic comparisons between malnourished and well-nourished OIPN patients, and multivariate models were at risk of overfitting. Consequently, our findings are exploratory and hypothesis-generating rather than confirmatory. Future longitudinal studies incorporating repeated metabolomic and nutritional assessments in larger, more balanced cohorts are essential to establish temporal relationships, elucidate causal pathways, and directly investigate how nutritional status modulates the metabolic underpinnings of OIPN.

In addition, among the differentially expressed metabolites, lipids exhibited the second-most prominent alterations (Figure 3E). Lipid metabolism is known to be involved in tumorigenesis and disease progression in various cancers, including colorectal cancer, through modifications to lipid synthesis, storage, and catabolism. Previous studies have shown that lipid alterations may arise as a consequence of cancer treatment and could contribute to treatment resistance (29). However, the specific role of lipid metabolism in OIPN pathogenesis among CRC patients remains unclear and warrants further investigation.

This study has delineated the metabolomic alterations associated with OIPN in colorectal cancer patients, both in the overall cohort and when stratified by nutritional status. By integrating clinical nutritional assessment with deep metabolomic profiling, we provide novel insights into the metabolic mechanisms underlying OIPN and highlight the significant influence of malnutrition on its development. However, these findings are exploratory and should be interpreted with caution, given the study’s limitations. Targeted nutritional support interventions cannot be recommended based on the current data alone. Future research—including longitudinal studies with repeated metabolomic and nutritional assessments, larger and more balanced cohorts, and mechanistic experiments in preclinical models—is essential to validate these findings, establish causal relationships, and determine whether modulation of specific metabolic pathways could serve as a therapeutic strategy for OIPN prevention or management.

## Data Availability

The raw data supporting the conclusions of this article will be made available by the authors, without undue reservation.

## Footnotes

1
http://www.hmdb.ca

2
http://www.massbank.jp/

3
https://www.genome.jp/kegg/

4
http://www.lipidmaps.org

5
https://www.mzcloud.org
